# E-selectin and vascular cell adhesion molecule-1 as biomarkers of 3-month outcome in cerebrovascular diseases

**DOI:** 10.1186/s12950-015-0106-z

**Published:** 2015-11-04

**Authors:** Sébastien Richard, Linnéa Lagerstedt, Pierre R. Burkhard, Marc Debouverie, Natacha Turck, Jean-Charles Sanchez

**Affiliations:** Department of Neurology, Stroke Unit, Nancy University Hospital Center, 29 avenue du Marechal de Lattre de Tassigny-CO n° 34, 54035 Nancy, Cedex France; Department of Human Protein Sciences, University Medical Center, Rue Michel Servet 1, 1211 Geneva, Switzerland; Department of Neurology, Geneva University Hospitals and Faculty of Medicine, University of Geneva, Rue Gabrielle-Perret-Gentil 4, 1205 Geneva, Switzerland

**Keywords:** Neuro inflammation, Cell adhesion molecules, E-selectin, VCAM-1, Biomarkers, Cerebrovascular disease, Stroke, Outcome, Prognosis

## Abstract

**Background:**

Inflammation is known to worsen cerebral damage at the acute phase of stroke. In this setting, cell adhesion molecules (CAMs) play a crucial role mediating migration of immune cells into the infarcted area. However, their value in long-term outcome prediction for patients with cerebrovascular diseases (CVD) is less described.

**Methods:**

Levels of four CAMs (E-selectin, P-selectin glycoprotein ligand-1, intercellular adhesion molecule-1, and vascular cell adhesion molecule-1 (VCAM-1)) and six other known biomarkers (C-reactive protein (CRP), interleukin-6 (IL-6), N-terminal pro-brain natriuretic peptide (NT-proBNP), troponin I, vasopressin-neurophysin 2-copeptin, and S100 calcium-binding protein B) were measured in a population of patients presenting CVD. Blood collections for analysis were performed within different time windows after stroke onset: 0–6 h, 6–36 h, 2–3 days, 5–7 days, and 2–3 weeks. Independent associations with poor outcome at 3 months (modified Rankin Scale score > 2) were sought using univariate and multivariate analysis after adjustments for age and National Institute of Health Stroke Scale score. Predictive ability of each biomarker has also been assessed with ROC analysis.

**Results:**

One hundred patients were prospectively included whom 75 presented with ischemic strokes, nine with hemorrhagic strokes and 16 with transient ischemic attacks. During the first 6 h after stroke onset, E-selectin was found to be an independent predictor of 3-month outcome (odds ratio (OR) =24; 95 % confidence interval (95 % CI), 2–354; *p* = 0.022) (area under the curve (AUC) =78 %), as was VCAM-1 during the third week after onset (OR = 8; 95 % CI, 2–37; *p* = 0.01) (AUC = 73 %). Associations remained after the exclusion of patients with hemorrhagic strokes and transient ischemic attacks. Independent associations with outcome were also found for CRP (OR = 5; 95 % CI, 1–22; *p* = 0.023) and IL-6 (OR = 5; 95 % CI, 1–17; p = 0.021) at 2–3 days and for NT-proBNP at 6–36 h (OR = 20; 95 % CI, 1–337; *p* = 0.04).

**Conclusions:**

E-selectin and VCAM-1 were independent predictors of outcome in a population of patients with CVD. The predictive capability of other biomarkers known to be indicators for prognosis also emerged, confirming the study’s robustness. CAMs levels could be considered as objective biological criteria for prognosis in CVD.

**Electronic supplementary material:**

The online version of this article (doi:10.1186/s12950-015-0106-z) contains supplementary material, which is available to authorized users.

## Background

For physicians in stroke units to provide the most appropriate care, they need to be able to predict outcome in patients with cerebrovascular diseases (CVD). At different stages in stroke management, outcome prediction helps in decision making about reperfusion therapies, discriminating between patients needing greater monitoring, and even the difficult choice between intensive care measures and withholding treatment. Initial clinical scores, like the National Institute of Health Stroke Scale (NIHSS), are reliable tools. However, adding objective paraclinical parameters to accurate outcome prediction techniques has remained a challenge [1]. Finding blood biomarkers independent of other known predictors of outcome could lead to objective biological criteria applicable to future prognosis scores. Inflammation is a consistent reaction of cerebrovascular events. At the acute phase, it leads to brain swelling and extension of ischemic area [2]. For this reason, its effect on prognosis is of high interest. Association with outcome in CVD has been widely described for some proteins related to the inflammatory pathway, especially interleukin-6 (IL-6) and C-reactive protein (CRP) [3, 4]. Cell adhesion molecules (CAMs) play a crucial role to initiate inflammatory mechanisms soon after cerebral damage, not only during ischemia [5], but also during hemorrhagic strokes [6]. They promote migration of immune cells across the blood–brain barrier within the cerebral parenchyma [7]. However, little is known about their predictive ability in CVD. The value of measuring E-selectin and vascular cell adhesion molecule-1 (VCAM-1) has only been described for predicting nearest evolution of stroke during acute phase, but it has never been assessed for long-term outcomes [8]. The aim of this study is to assess, at different time points during the management of a population presenting CVDs, whether CAMs can predict outcome at 3 months. To back up our results, we concurrently assessed biomarkers with predictive value previously demonstrated in the literature, including CRP and IL-6 [9].

## Methods

Recruitment for this prospective cohort study took place at Geneva University Hospital’s emergency unit in Switzerland during a period of 2 years.

### Patients

For inclusion, patients had to be admitted within 36 h of the onset of stroke or transient ischemic attack (TIA). Diagnosis had to be corroborated by a neurologist’s investigation and cerebral imaging (magnetic resonance imaging or a computed tomography scan). Exclusion criteria were pregnancy, being a minor, presenting with a stroke with an undetermined time of onset, cancer, liver cirrhosis, renal failure, recent myocardial infarction (less than 3 months previously), and ongoing treatment with neuroleptics or lithium. Inclusions were not consecutive but were dependent on the presence of the paramedical staff in charge of the study (from Monday to Friday during usual working hours). Outcome was assessed at 3 months with modified Rankin Scale (mRS) score by a neurologist.

### Blood collection

For each included patient, blood samples were obtained within the following time windows after onset of the cerebral event: within the first 36 h, between 48 and 72 h (2–3 days), between days 5 and 7 (5–7 days), and during the third week (2–3 weeks). In the earliest time window, the results of blood samples drawn before and after the sixth hour following stroke onset were dichotomized to create time windows at 0–6 h and 6–36 h. The first blood sample was drawn before any reperfusion therapy.

### Biomarker measurements

Four CAMs were quantified in each blood sample: E-selectin, P-selectin glycoprotein ligand-1 (P-selectin), intercellular adhesion molecule-1 (ICAM-1), and VCAM-1. The levels of six other biomarkers with known predictive values were also assessed: CRP, IL-6, N-terminal pro-brain natriuretic peptide (NT-proBNP), troponin I, vasopressin-neurophysin 2-copeptin (copeptin), and S100 calcium-binding protein B (S100B). All measurements were performed using immunoassays, as has been previously described [10], except for copeptin, which was quantified using the US KRYPTOR compact PLUS^®^ (Thermo Scientific, BRAHMS, Germany). All measurements were blinded.

### Statistical analysis

All analyses were carried out using IBM SPSS Statistics software, version 20 (SPSS Inc., Chicago, IL, USA). The continuous variables described were frequency and mean ± standard deviation, along with categorical factors such as frequencies and percentages. The patients were dichotomized into good (mRS ≤ 2) or poor (mRS > 2) outcomes. First comparisons between the clinical characteristics and levels of each protein in each group were carried out at different times using Mann–Whitney, Chi-Square, and Fischer exact tests as appropriate, with significance set at *p* < 0.05. A Bonferroni correction was used as a *post hoc* test when a protein obtained a significant result, in order to adapt the *p*-values by the number of biomarkers (*p*-value/10). For proteins showing significantly different levels between groups, the continuous characteristic was expressed as a binary factor using the best cut-off for maximizing sensitivity and specificity according to the Youden index criterion (receiver operating characteristic analysis). For each biomarker with a poor outcome, an independent association was sought using univariate and multivariate logistic regressions, after adjustment for the clinical characteristics found to be statistically different during the first comparison. Next, potential independent associations between CAMs and outcomes, found in the overall population, were sought using three subgroups: subgroup 1 of patients presenting with ischemic events only (excluding hemorrhagic strokes); subgroup 2 of patients with cerebral damage only (excluding TIA); and subgroup 3 of patients with ischemic strokes only (excluding TIA and hemorrhagic strokes).

### Ethics

This study was approved by the local ethics committee N.A.C. (Neuclid, Apsic, Chirurgie department) of the Geneva University Hospitals, as study CER05–026 (05–058). It was carried out in accordance with the Declaration of Helsinki principles. Each patient, or a legally authorized representative, was informed about the study and gave consent to participate.

## Results

**Fig. 1:**
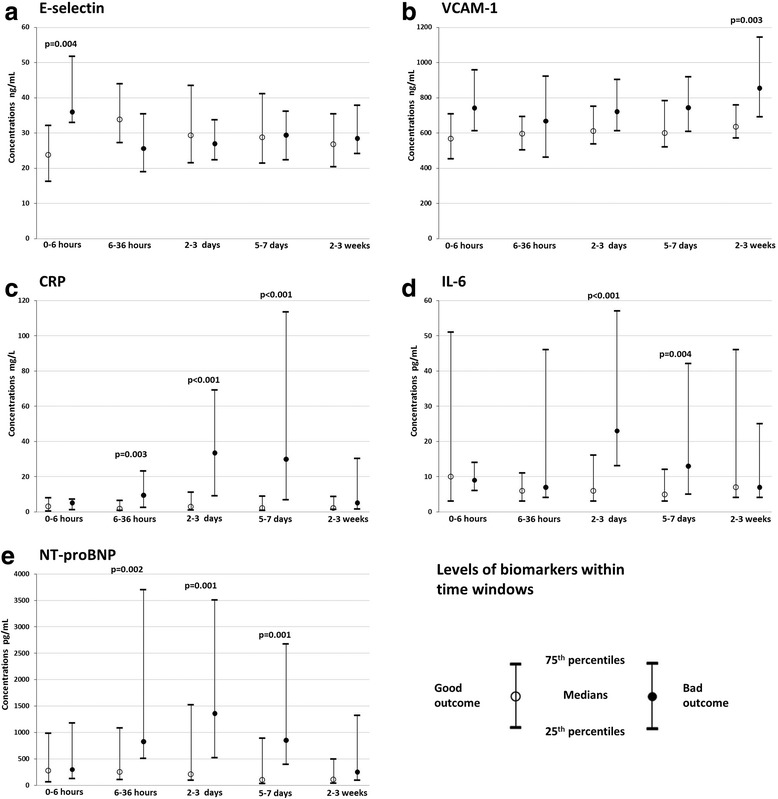
Levels of biomarkers within the different time windows according to patients’ outcome. Levels of E-selectin (**a**), vascular cell adhesion molecule-1 (**b**), C-reactive protein (**c**), interleukin-6 (**d**), and N-terminal pro-brain natriuretic peptide (**e**) are described as median, 25 and 75th percentiles; difference between levels considered as significant for *p* < 0.005 (after Bonferroni correction)

**Fig. 2:**
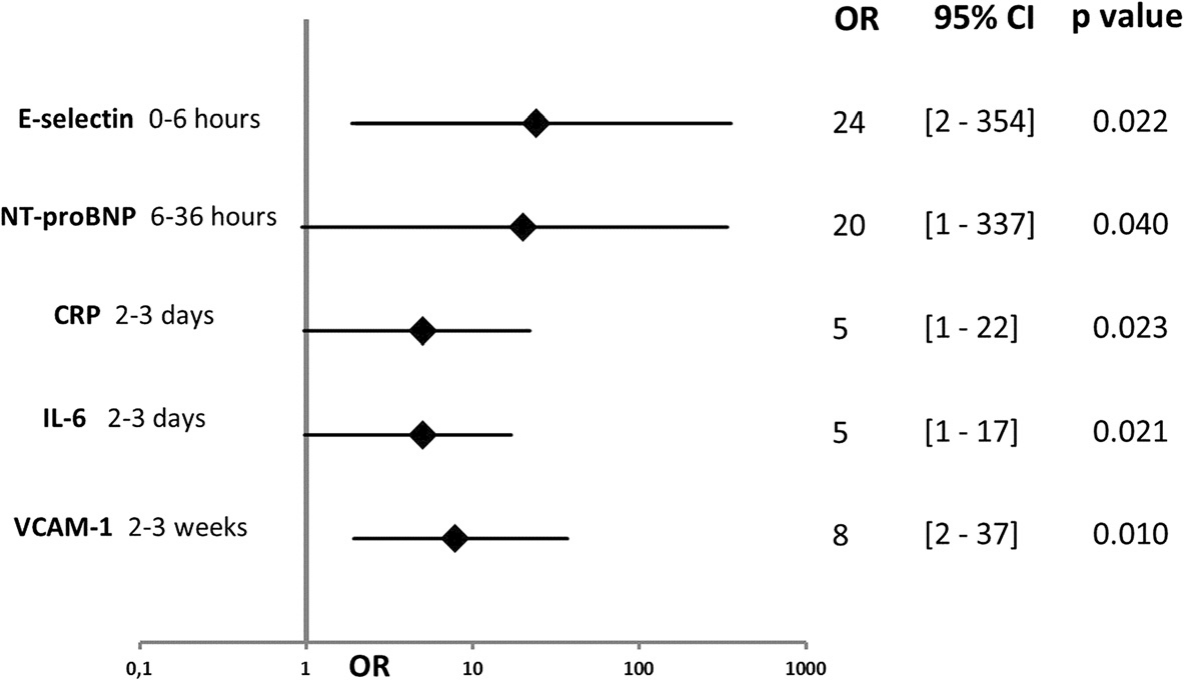
Biomarkers significantly associated with 3-month poor outcome in multivariate analysis. Each biomarker separately adjusted for age and National Institute of Health Stroke Scale at admission; OR, odds ratio; 95 % CI, 95 % confidence interval

**Fig. 3:**
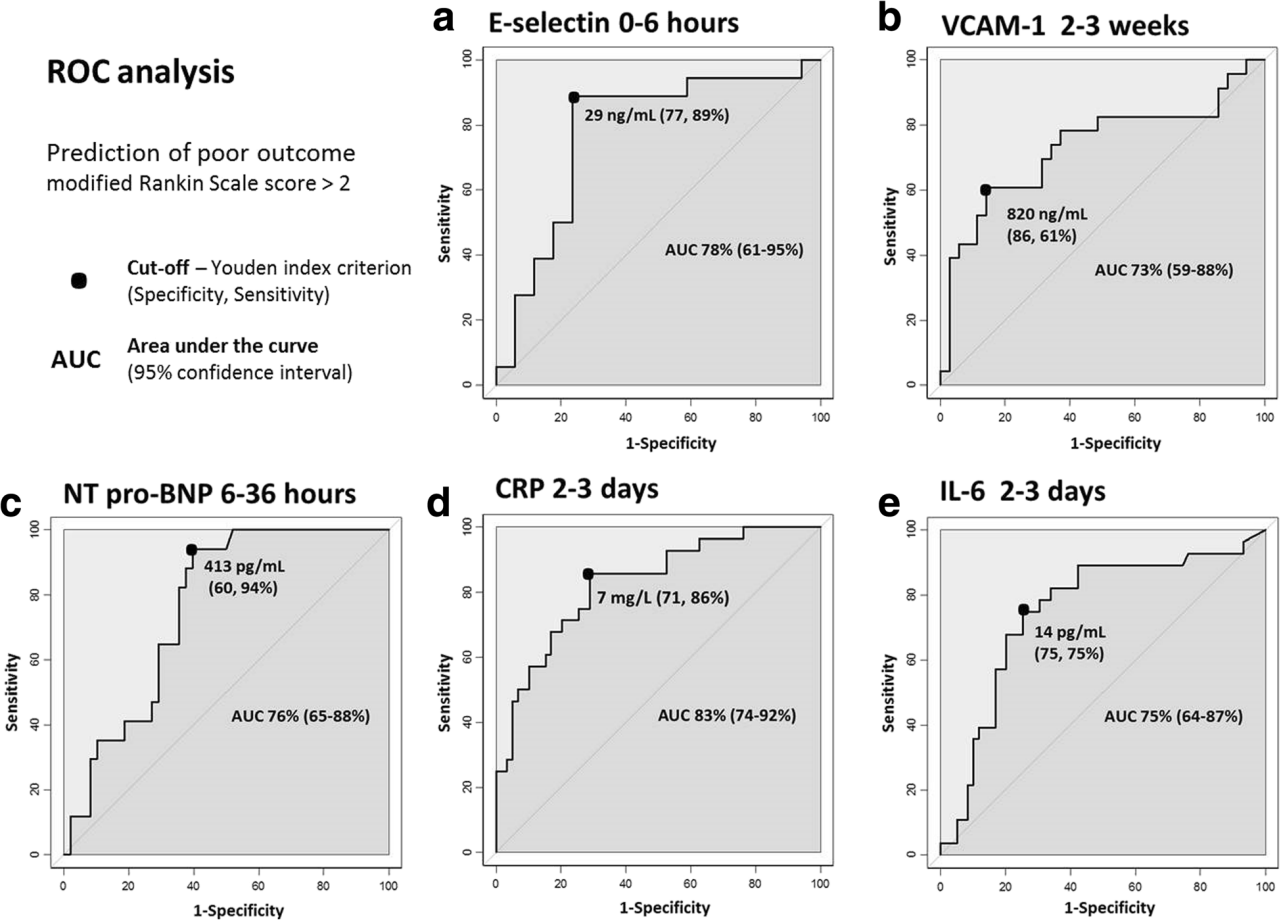
ROC analysis for biomarkers to predict 3-month poor outcome in patients with cerebrovascular diseases. Results for E-selectin at 0–6 h (**a**), vascular cell adhesion molecule-1 at 2–3 weeks (**b**), N-terminal pro-brain natriuretic peptide at 6–36 h (**c**), C-reactive protein at 2–3 days (**d**), and interleukin-6 at 2–3 days (**e**) are described as cut-off determined with Youden index criterion, respective specificity and sensitivity, area under the curve (AUC) and 95 % confidence interval; poor outcome defined as modified Rankin Scale score > 2

One hundred patients were included in this study, of whom 75 presented with ischemic strokes, nine with hemorrhagic strokes, and 16 with TIA. A good outcome was observed in 65 % of these patients. The population’s clinical characteristics and comparisons according to outcome are presented in the Table 1. Age (*p* = 0.007) and NIHSS score at admission (*p* < 0.001) were significantly higher in patients with a poor outcome. An initial blood sample was drawn from 35 patients during the first 6 h following onset. Of the 10 proteins measured, E-selectin, VCAM-1, CRP, IL-6, NT-proBNP, and S100B were at significantly higher levels in the group with a poor outcome in one of the different measuring times (Table 2, Fig. 1). After multivariate analysis, with adjustments made for age and NIHSS score at admission, expected independent associations with a poor outcome did indeed emerge for the known predictors of poor outcome: CRP (odds ratio (OR) =5; 95 % confidence interval (95 % CI), 1–22; *p* = 0.023) (area under the curve (AUC) =83 %) and IL-6 (OR = 5; 95 % CI, 1–17; *p* = 0.021) (AUC = 75 %) at 2–3 days, and NT-proBNP (OR = 20; 95 % CI, 1–337; *p* = 0.04) (AUC = 76 %) at 6–36 h (Figs. 2, 3).

**Table 1:** Baseline clinical characteristics of the population with comparison according to outcome

Characteristics	Total population	Good outcome	Poor outcome	*p* value
*n*	100	65	35	
Hemorrhagic stroke *n* (%)	9 (9)	3 (4.6)	6 (17.1)	
Ischemic stroke *n* (%)	75 (75)	46 (70.8)	29 (82.9)	
TIA *n* (%)	16 (16)	16 (24.6)	0 (0)	
Age mean ± SD, years	66 ± 15	63 ± 15	72 ± 13	0.007^a^
Male *n* (%)	55 (55)	40 (61.5)	15 (42.9)	0.073^b^
NIHSS score mean ± SD	7.91 ± 6.42	5 ± 4	14 ± 5	<0.001^a^
SBP mean ± SD, mmHg	154 ± 29	151 ± 29	160 ± 28	0.101^a^
DBP mean ± SD, mmHg	85 ± 15	85 ± 14	85 ± 16	0.960^a^
Hypertension *n* (%)	63 (63)	39 (60)	24 (68.6)	0.397^b^
Dyslipidemia *n* (%)	36 (36)	26 (40)	10 (28.6)	0.256^b^
Diabetes *n* (%)	13 (13)	6 (9.2)	7 (20)	0.210^c^
Tobacco *n* (%)	43 (43)	30 (46.2)	13 (37.1)	0.385^b^
Alcohol *n* (%)	2 (2)	0 (0)	2 (5.7)	0.120^c^
Oral contraceptive *n* (%)	1 (1)	1 (1.5)	0 (0)	1.000^c^
Migraine *n* (%)	4 (4)	4 (6.2)	0 (0)	0.295^c^
Atrial fibrillation *n* (%)	33 (33)	18 (27.7)	15 (42.9)	0.124^b^
Coronary disease *n* (%)	4 (4)	3 (4.6)	1 (2.9)	1.000^c^

*TIA* transient ischemic attack, *NIHSS* National Institute of Health Stroke Scale, *SBP/DBP* systolic/diastolic blood pressure, *SD* standard deviation

^a^Mann–Whitney *U* test

^b^Chi-Square test

^c^Fischer exact test

**Table 2:** Biomarkers with significant higher levels in patients with 3-month poor outcome, univariate and ROC analysis

Biomarker	Comparison mean concentrations+/−SD			ROC curves		Univariate analysis	
Time	Good outcome	Poor outcome	*p* ^a^	AUC (95 % CI)	Cut-off	OR (95 % CI)	*p* ^b^
E-selectin							
0–6 h	26 ± 15 ng/mL	41 ± 19 ng/mL	0.004	78 % (61–95)	29 ng/mL	26 (4–165)	0.001
VCAM-1							
2–3 weeks	669 ± 197 ng/mL	886 ± 284 ng/mL	0.003	73 % (59–88)	820 ng/mL	9 (3–33)	0.001
CRP							
6–36 h	6 ± 13 mg/L	18 ± 26 mg/L	0.003	75 % (62–88)	5 mg/L	6 (2–18)	0.005
2–3 days	9 ± 15 mg/L	54 ± 68 mg/L	<0.001	83 % (74–92)	7 mg/L	15 (4–49)	<0.001
5–7 days	15 ± 53 mg/L	72 ± 101 mg/L	<0.001	81 % (71–91)	9 mg/L	9 (3–26)	<0.001
IL-6							
2–3 days	50 ± 209 pg/mL	193 ± 819 pg/mL	<0.001	75 % (64–87)	14 pg/mL	9 (3–25)	<0.001
5–7 days	70 ± 274 pg/mL	21 ± 20 pg/mL	0.004	70 % (58–82)	4 pg/mL	12 (3–54)	0.002
NT-proBNP							
6–36 h	1135 ± 2215 pg/mL	2547 ± 3286 pg/mL	0.002	76 % (65–88)	413 pg/mL	24 (3–200)	0.003
2–3 days	1254 ± 2235 pg/mL	3899 ± 7161 pg/mL	0.001	73 % (62–84)	314 pg/mL	18 (4–82)	<0.001
5–7 days	1134 ± 2736 pg/mL	2055 ± 2557 pg/mL	0.001	73 % (62–84)	272 pg/mL	9 (3–26)	<0.001
S100B							
2–3 days	31 ± 57 pg/mL	336 ± 566 pg/mL	0.003	70 % (57–83)	30 pg/mL	5 (2–13)	0.001

*SD* standard deviation, *ROC* receiver operating characteristic analysis, *AUC* area under the curve, *OR* odds ratio, *95 % CI* 95 % confidence interval; poor outcome defined as modified Rankin Scale score > 2

^a^Comparison of biomarkers levels between both groups, good and poor outcome, with Mann–Whitney *U* test, level of significance with Bonferroni correction (*p* < 0.005)

^b^Univariate analysis performed for biomarkers after determination of cut-off using ROC analysis

**Fig. 4:**
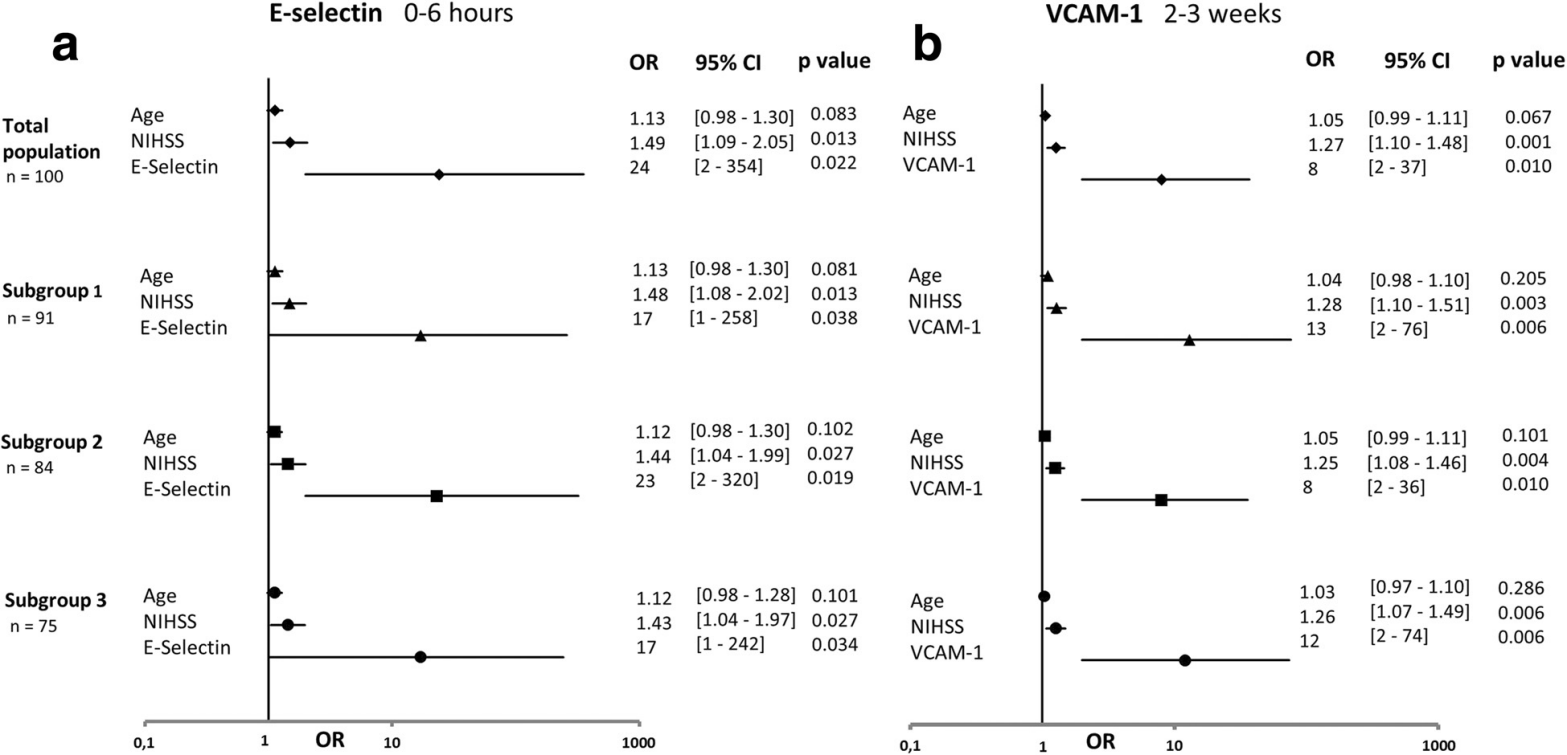
Multivariate analysis for association of E-selectin and vascular cell adhesion molecule-1 with 3-month poor outcome. Results in the total population and subgroups; time windows 0–6 h for E-selectin (**a**), and 2–3 weeks for vascular cell ahesion molecule-1 (**b**); each biomarker separately adjusted for age and National Institute of Health Stroke Scale (NIHSS) at admission; OR, odds ratio; 95 % CI, 95 % confidence interval; subgroup 1: patients with ischemic events, i.e. excluding hemorrhagic strokes; subgroup 2: patients with cerebral damage, i.e. excluding transient ischemic attacks; subgroup 3: patients with ischemic strokes, i.e. excluding hemorrhagic strokes and transient ischemic attacks

More interestingly, after multivariate analysis, E-Selectin was found to be an early independent predictor of poor outcome at 0–6 h at levels > 29 ng/mL (OR = 24; 95 % CI, 2–354; *p* = 0.022) (AUC = 78 %), whereas VCAM-1 was a predictor during the 2–3 week measurement at levels > 820 ng/mL (OR = 8; 95 % CI, 2–37; *p* = 0.01) (AUC = 73 %) (Table 2, Figs. 2, 3). These independent associations for E-selectin and VCAM-1 were maintained within the three subgroups after adjustment for age and NIHSS (Fig. 4). Fourteen patients with ischemic stroke were treated with recombinant tissue plasminogen activator, of whom only four presented with a good outcome. There is no modification of the model after exclusion of these patients (data not shown). In view of these results about inflammatory biomarkers, we were interested in levels of matrix metalloproteinases (MMP). We did not find any significant rise of MMP-1 and MMP-3 levels between different times and any significant difference between both patient groups (Additional file 1).

## Discussion

Inflammation is known to worsen cerebral damage at the acute phase of stroke. The first step is represented by the release of pro inflammatory cytokines from necrotic cells like interleukin-1β (IL-1β) and tumor necrosis factor (TNF-α). The direct results are activation of the microglia and modification of permeability of the blood–brain barrier [11]. Endothelial cells of the arterial wall lose their tight junction and express CAMs [12]. These later permit recruitment and migration of leukocytes into the brain parenchyma [13]. Infiltration of cerebral tissue with macrophages, neutrophil granulocytes and T-cells has been demonstrated at different times following experimental stroke [14]. First, adherence of leukocytes to the arterial wall would contribute to microvessel obstruction [15]. More importantly, immune cells release reactive oxygen species and MMP leading to cell death, and cytokines maintaining inflammation [11]. This results in expansion of the infarction from the penumbra and brain swelling. On the other hand, macrophages permit clearance of necrotic debris and tissue repair by production of tropic factors during later phases [2].

In this study, the early predictive capability of E-selectin was specifically observed during the key period of the first 6 h after stroke onset—the period during which questions about reperfusion therapy arise. VCAM-1 was an indicator of prognosis in a later phase of patient management, with a significant increase at 2–3 weeks. Both CAMs mediate adhesion of the monocytes and granulocytes that participate in the development of cerebral edema [7], whatever the ischemic or hemorrhagic feature of cerebral damage [5, 6]. E-selectin first recruits leukocytes rolling on the vessel wall, whereafter a stronger adhesion is mediated by VCAM-1 [15]. This could explain their correlation with prognosis at the acute phase of stroke, early cytotoxic and vasogenic edema being responsible for both the extension of the ischemic area and rising intracranial hypertension [5]. They are both expressed by endothelial cells, and VCAM-1 is also found in smooth muscle cells [5, 16]. Expression of E-selectin and VCAM-1 seems not to be concurrent in our study. A first phase of CAMs synthesis occurs soon after stroke onset, subsequent to vascular damage and activation of the arterial wall [5, 17]. In vitro and in vivo studies show E-selectin is the first to be released by activated endothelial cells during the first 24 h with rapid decrease [5, 18]. This permits initial recruitment of granulocytes and monocytes by rolling on activated endothelium. High levels of VCAM-1 are found later in the blood and remain for several days [5]. This corresponds to migration of monocytes within brain parenchyma through VCAM-1. Such high levels of E-selectin and VCAM-1 have been described as decreasing within the first 4 days after stroke onset, in association with the clinical improvements seen in patients [8]. A second endothelial activation leads to the production of CAMs at the end of the second week after stroke. This production could participate in tissue remodeling, depending on the volume of cerebral necrosis [5]. We observe at this time high levels of VCAM-1 only, which could in part explain its performance as a prognosis indicator during the third week in our study. Moreover, VCAM-1 is suspected to mediate inflammation in atherosclerotic plaques, increasing the risk of rupture and a recurrence of vascular events in stroke patients [19]. It would also be involved in the arterial wall disease of cerebral lacunar infarctions due to hypertension [20]. During secondary analysis, we failed to find any rise of MMP. These proteins are released by recruited leukocytes and are implicated in brain damage including blood–brain barrier disruption [21, 22]. However, we disposed of MMP-1 and 3 concentrations only. Study of MMP-2 and 9, more described in the cerebrovascular field, would be more relevant in future works [23, 24].

The reliability of the present findings is supported by the fact that the study confirms the predictive capability of known biomarkers. During the third day following stroke onset, two well-known proteins of the inflammatory pathway—CRP and IL-6—were found to be indicators of prognosis in our population. CRP is generated in the liver, but it is also found in smooth muscle cells, with a potential role in the growth and the rupture of atherosclerotic plaques [25, 26]. Elevated levels of CRP have been described from 3 h after stroke onset, with a plasmatic peak between 36 and 48 h, and an ability to predict outcome lasting several days [3]. Although IL-6 is a ubiquitous cytokine, it has been demonstrated that its production is higher within 72 h of stroke onset, and it has a key role at this stage of inflammatory response. Production of IL-6 is induced by TNF-α and IL-1β, leading to a synthesis of CRP, fibrinogen and CAMs, activation of the hypothalamic-pituitary-adrenal axis, and fever. CRP and IL-6 represent a further step along the inflammatory pathway, promoting disruption of the blood–brain barrier, edema, and vascular thrombosis [4]. They can also indicate a state of infection, a condition that worsens the prognosis for patients with stroke. As a result, both are already widely described as predictors of poor outcome, mortality, and severity, including in cases of ischemic [27, 28] or hemorrhagic strokes [29].

The NT-proBNP cardiac biomarker is the result of the body metabolizing BNP—a vasoactive hormone secreted by the ventricular myocardium subsequent to wall stress and the stretching of myocytes. BNP plays a role in the regulation of blood pressure via natriuretic, diuretic, and vasodilatory effects. Higher levels are observed after the first 24 h following stroke onset, especially in patients with cardiac diseases like left ventricular failure and arrhythmia, which are responsible for greater mortality and the more serious, larger cerebral infarctions [30, 31]. NT-proBNP is considered as a predictor of cardioembolic etiology [32], poor outcome [33], recurrence [16], and mortality [34]. This biomarker can predict both evolution during the acute phase and long term prognosis, and it was found consistently in our results in the 6–36-h period [35].

S100B is a calcium-binding protein found in the central nervous system. Its presence in circulating blood is an indication of disruption at the blood–brain barrier. It is recognized as a biomarker of prognosis in stroke until 6 days after the event, resulting from its strong correlation with infarction size and severity [36]. However, after adjustment for age and NIHSS score, we failed to find independent associations of S100B with poor outcome.

One of this study’s aims was to select biomarkers predicting outcome in a population of patients combining the most usual types of CVD. Indeed, in clinical practice, a first blood sample is drawn early on during admission of patients in order for urgent tests to be made, before cerebral imaging and assessment of the final diagnosis. This would be the appropriate time for a first measurement of biomarkers, hence the interest in a common indicator of prognosis, whatever the type of CVD. Our study included patients with different types of CVDs, with a distribution comparable to that encountered in stroke units. However, the validity of determining biomarkers for predicting prognosis in a heterogeneous population combining CVDs of such different physiopathology seemed questionable. We thus further validated our results in subgroups with specific types of CVDs. E-selectin and VCAM-1 remained independent predictors of outcome in all three subgroups, as they did in the overall population. An appropriate analysis could not be performed on the subgroup of patients presenting with hemorrhagic strokes because the sample size was too small.

Furthermore, we studied large time windows instead of specific endpoints; this was in order to help physicians who need to assess a prognosis during periods representing the different steps of a patient’s management [37]. For instance, time of admission of patients with stroke can vary widely, from the very acute phase, when thrombolysis can be considered, until many hours after onset for those patients who consult a physician following a TIA or minor deficits.

This study has some limitations. Collection of data by paramedical staff in non-consecutive cases resulted in a lack of important information like evolution of NIHSS score after the acute phase. The study’s limited sample size did not allow us to estimate the added utility of E-selectin and VCAM-1 measurement to predict outcome using net reclassification improvement (NRI) and integrated discrimination improvement (IDI). However, multivariate analysis with adjustments for age and NIHSS scores was used to select biomarkers presenting the greatest correlations with outcome, and ROC analysis to assess their predictive ability. Face with widespread values of proteins concentrations in the population, we chose to determinate a cut-off for each biomarker to assess association with outcome. This method is more prone to overestimation of biomarker accuracy, all the more in a limited sample size [38]. Future studies should assess the predictive capability of CAMs in a population with a homogenous initial clinical state. This could identify, for example, patients at a high risk of a worsening condition in cases of minor deficits, or, on the other hand, patients with serious strokes who are likely to improve. Such studies would require higher numbers of patients in multiple centres.

## Conclusions

The E-selectin and VCAM-1 CAMs were found to be independent predictors of outcome in a population of patients with CVD. The predictive capability of E-selectin is valid during the first 6 h following a stroke, while that of VCAM-1 is valid between the second and third week. These biomarkers could be considered as objective criteria and added to the clinical parameters of future scores for prognosis of stroke.

## Additional file

Additional file 1:
**Levels of matrix metalloproteinases within the different time windows according to patients’ outcome.** Levels of matrix metalloproteinase 1 (a), and matrix metalloproteinase 3 (b) are described as median, 25th and 75th percentiles; empty dots: good outcome patient group, black-filled dots: bad outcome patient group. (TIFF 88 kb)

## Abbreviations

95 % CI: 95 % confidence interval
AUC: Area under the curve
CAMs: Cell adhesion molecules
Copeptin: Vasopressin-neurophysin 2-copeptin
CRP: C-reactive protein
CVD: Cerebrovascular diseases
ICAM-1: Intercellular adhesion molecule-1
IL-1β: Interleukin-1β
IL-6: Interleukin-6
MMP: Matrix metalloproteinase
mRS: Modified Rankin scale
NIHSS: National Institute of Health Stroke Scale
NT-proBNP: N-terminal pro-brain natriuretic peptide
OR: Odds ratio
P-selectin: P-selectin glycoprotein ligand-1
ROC: Receiver operating characteristic
S100B: S100 calcium-binding protein B
TIA: Transient ischemic attack
TNF-α: Tumor necrosis factor
VCAM-1: Vascular cell adhesion molecule-1
