# Supplementary figures and images for: E-selectin and vascular cell adhesion molecule-1 as biomarkers of 3-month outcome in cerebrovascular diseases
Source: J Inflamm (Lond). 2015 Nov 4;12:61. doi: 10.1186/s12950-015-0106-z (PMC4634720; doi:10.1186/s12950-015-0106-z)

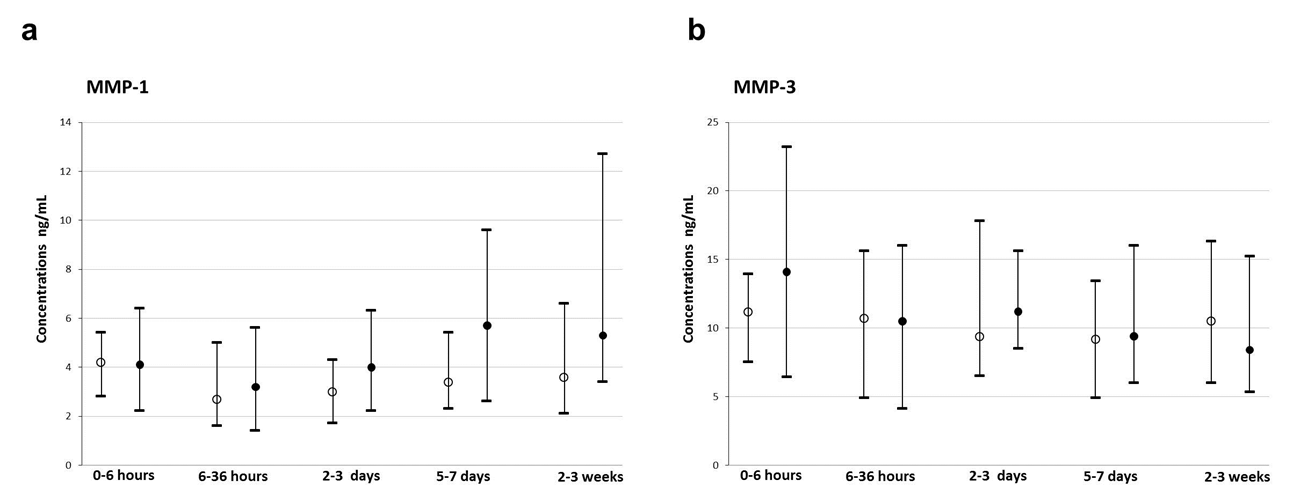

Supplement: Additional file 1: — Levels of matrix metalloproteinases within the different time windows according to patients’ outcome. Levels of matrix metalloproteinase 1 (a), and matrix metalloproteinase 3 (b) are described as median, 25th and 75th percentiles; empty dots: good outcome patient group, black-filled dots: bad outcome patient group. (TIFF 88 kb) [file 12950_2015_106_MOESM1_ESM.tif]
